# Enhanced CHOLESTEROL biosynthesis promotes breast cancer metastasis via modulating CCDC25 expression and neutrophil extracellular traps formation

**DOI:** 10.1038/s41598-022-22410-x

**Published:** 2022-10-17

**Authors:** Qiqi Tang, Beibei Liang, Lisha Zhang, Xuhui Li, Hengyu Li, Wei Jing, Yingjie Jiang, Felix Zhou, Jian Zhang, Yanchun Meng, Xinhua Yang, Hao Yang, Gang Huang, Jian Zhao

**Affiliations:** 1 Shanghai Key Laboratory of Molecular Imaging, Shanghai University of Medicine and Health Science, 279Th Zhouzhu Road, Shanghai, 201318 China; 2 Shanghai Key Laboratory of Molecular Imaging, Jiading District Central Hospital Affiliated Shanghai University of Medicine and Health Sciences, Shanghai, 201318 China; 3 Shanghai University of Traditional Medicine, Shanghai, 201203 China; 4 Changhai Hospital, Navy Military Medical University, Shanghai, 200438 China; 5 Ludwig Institute for Cancer Research, Nuffield Department of Clinical Medicine, University of Oxford, Oxford, OX3 7DQ UK; 6 Phase I Clinical Trial Center, Shanghai Cancer Center, Fudan University, Shanghai, 200032 China; 7 Department of Oncology, Shanghai Medical College, Fudan University, Shanghai, 200032 China

**Keywords:** Cancer, Cell biology, Immunology, Oncology

## Abstract

Neutrophil extracellular traps (NETs) has been demonstrated to regulate the metastasis of breast cancer. In this study, we showed that de novo cholesterol biosynthesis induced by ASPP2 depletion in mouse breast cancer cell 4T1 and human breast cancer cell MDA-MB-231 promoted NETs formation in vitro, as well as in lung metastases in mice intravenously injected with ASPP2-deficient 4T1 cells. Simvastatin and berberine (BBR), cholesterol synthesis inhibitors, efficiently blocked ASPP2-depletion induced NETs formation. Cholesterol biosynthesis greatly enhanced Coiled-coil domain containing protein 25 (CCDC25) expression on cancer cells as well as in lung metastases. CCDC25 expression was co-localized with caveolin-1, a lipid raft molecule, and was damped by inhibitor of lipid rafts formation. Our data suggest that cholesterol biosynthesis promotes CCDC25 expression in a lipid raft-dependent manner. Clinically, the expression of CCDC25 was positively correlated with the expression of 3-hydroxy-3-methylglutaryl-CoAreductase (HMRCG), and citrullinated histone H3 (H3cit), in tissues from breast cancer patients. High expression of CCDC25 and HMGCR was related with worse prognosis in breast cancer patients. In conclusion, our study explores a novel mechanism for de novo cholesterol biosynthesis in the regulation of CCDC25 expression, NETs formation and breast cancer metastasis. Targeting cholesterol biosynthesis may be promising therapeutic strategies to treat breast cancer metastasis.

## Introduction

Cholesterol is generated through mevalonate (MVA) pathway by a series of enzymatic steps^1^. HMGCR is the rate-limiting enzyme in MVA pathway, whose expression is tightly regulated by transcription factor sterol regulated element binding protein-2 (SREBP-2) and can be blocked by statins. Cholesterol is generally considered as a risk factor for breast cancer^2,3^. Cholesterol is the obligatory precursor of steroid hormones, which drive the initiation and promotion of breast cancer^4,5^. Cholesterol is also a fundamental structural component of mammalian cell membranes, needed by highly proliferative cancer cells^6^. Cholesterol also serves as an integral component of lipid rafts, which are critical to form transmembrane signaling complexes^7^. Though, epidemiologic data concerning the impact of cholesterol on breast cancer onset are still controversial^8–10^. Strong clinical evidence has supported the role for cholesterol in the recurrence and survival of breast cancer patients. Several retrospective studies have shown patients taking statins had reduced breast cancer recurrence^11–14^. Experimental data also support the importance for cholesterol in breast cancer metastasis. Cholesterol metabolite 27-hydroxycholesterol has been suggested to regulate breast cancer metastasis by remodeling tumor microenvironment, stimulating recruitment of immune cells like neutrophils and γδT-cells to the metastatic niche^15^. 27-hydroxycholesterol also enhanced resistance to ferroptosis, a feature of metastatic cells in cancer cells, through increasing the expression of the lipid peroxidase GPX4, a key negative regulator of ferroptotic cell death^16^. Cholesterol synthesis has been linked to increasing stemness of cancer cells, which is important for metastasis initiation^17,18^.

Neutrophils are abundant leukocytes in human peripheral blood as the first defender to against microorganisms. Recently, evidences have revealed that neutrophils are important components in tumor microenvironment, promoting breast cancer progression and metastasis^19,20^. Clinically, increased neutrophil abundance predicts poor prognosis in breast cancer patients^21,22^. Recent evidences show that NETs, web-like structures composed of granule proteins and decondensed chromatin, extruded by neutrophils play critical roles in promoting metastasis in breast cancer mouse models^23,24^. The importance of NETs in metastasis was further proved in breast cancer patients with metastases^25^. The way that NETs promote metastasis probably involved attracting cancer cells to distant metastatic organs, trapping of disseminated circulated cancer cells and awakening the dormancy of cancer cells^24–26^. The transmembrane protein CCDC25 was identified as the receptor on cancer cell surface to bind with surrounding NET-DNA promoting NETs formation. CCDC25-knockout in human breast cancer cells MDA-MB-231 abrogated NET-mediated lung metastases upon intravenous injection of cancer cells in LPS-stimulated mice or the formation of liver metastases upon intrasplenic injection. Clinically, high expression of CCDC25 on primary breast cancer cells was associated with reduced metastasis-free survival^25^. However, the roles of cholesterol in regulation of CCDC25 expression and NETs formation are largely unknown.

Enhanced cholesterol biosynthesis could be induced by various oncogenic signals in cancer cells, such as PI3K-AKT, mTORC1 and AMPK signals^27^. Cholesterol biosynthesis could also be regulated by oncogene or tumor suppresser genes, such as Myc^28^, mutant p53^29^, RB9^30^, and ASPP2^31^, by interaction with transcription factor SREBP-2, the master regulator of cholesterol biosynthesis^32^. We have previous demonstrated that ASPP2, a haploinsufficient tumor suppressor^33^, interacts with SREBP-2 in the nucleus and inhibits the expression of HMGCR^31^. Tumor cells showed higher HMGCR expression, elevated cholesterol levels, increased tumor-initiating capability by depletion of ASPP2. Simvastatin, a HMGCR inhibitor efficient reversed the stemness characteristics of ASPP2-depleted cells^31^. Previous study has proved the role of ASPP2 in regulating cell plasticity and metastasis^34^. Down-regulation of ASPP2 has been proved to promote epithelial to mesenchymal transition and enhance invasive capability of breast cancer cells.

Here we used lentivirus-mediated ASPP2 depletion to generate cancer cells with enhanced cholesterol biosynthesis in mouse breast cancer cell 4T1, and investigate the role of cholesterol in regulating CCDC25 expression, NETs formation and tumor metastasis in breast cancer.

## Materials and methods

### Cell culture

Mouse breast cancer cell 4T1 and human breast cancer cell MDA-MB-231 were cultured in RPMI-1640 (Hyclone) supplemented with 10% fetal bovine serum (FBS, Gibco), 100 U/ml penicillin and 100 µg/ml streptomycin (Invitrogen) at 37 °C in a humidified incubator containing 5% CO2.

### Lentivirus shRNA productions

Lentiviruses shRNA targeting mouse and human ASPP2 and HMGCR gene were designed and generated by Hanheng Biological Technology Co., Ltd (Shanghai, China). Details about lentivirus shRNA productions and lentivirus infection can be found in the supplementary data.

### Cholesterol content assay

For measurement of total and cholesterol concentration, the culture medium was replaced with serum-free medium and added with 2 μM Simvastatin (Sigma), 10 μM BBR (MCE) or control for 24 h, and then collected at least 5 × 10^6^ cells. The total or free cholesterol is measured by using Micro Total Cholestenone (Solarbio, Beijing, China) or free Cholestenone Content Assay Kit (Solarbio). The results are normalized based on the number of cells.

### Proliferation assay

For the colony formation assay, 2 × 10^3^ cells were seeded into 6-well plant and cultured for 24 h. Then LV-shASPP2 cells were treated with 2 μM simvatatin or 10 μM BBR for 24 h, after that the culture medium was replaced with medium containing 20% FBS for 10 days. Cell colonies were fixed with 4% paraformaldehyde (PFA) and stained with 0.1% crystal violet (Solarbio). The results presented are averages from three independent experiments.

### Sphere formation assay

7.5% bovine serum albumin, 4 μg/mL insulin (Sigma), 20 ng/mL basic fibroblast growth factor (PeproTech), B27 (1:50; Invitrogen), and 20 ng/mL epidermal growth factor (PeproTech,) were supplemented to DMEM-F12 medium. 4T1 cells were suspended at a concentration of 2000 cells/ml and 2 × 10^3^ cells were plated onto each well of ultralow attachment plates (Corning). On days 6, sphere numbers were counted as primary spheres using a Leica microscope. The spheroids were then pipetted with PBS to make single cells, re-suspended in the above-mentioned medium. After 8 days of incubation, sphere numbers were counted as secondary spheres.

### Invasion and NET information assays

1 × 10^5^ cancer cells in serum-free medium were add to rehydrated Matrigel (Corning) in the upper chamber. RPMI-1640 medium containing 20% FBS or neutrophils were added in the lower chamber. Neutrophils were freshly isolated from mice (Details can be found in the supplementary data), adjusted to a concentration of 5 × 10^5^ cells/ml, plated on the slides coated with poly-L-lysine-coated coverslips, stimulated with Phorbol 12-myristate 13-acetate (PMA, 20 nM, Sigma-Aldrich) in serum free RPM1I-1640. After 22 h in 37 °C, the trans-well chamber was fixed and stained with crystal violet. Counted the number of invaded cells and calculated the average number of cells in six fields randomly selected under an optical microscope.

The slides with neutrophils in the lower chamber were fixed with 4% PFA, permeabilized with 0.5% Triton X-100 (Sigma), and blocked with 1% bovine serum albumin (beyotime, Shanghai, China) for 2 h in room temperature. Incubated with anti-H3cit (1:400, Cell Signaling Technology) and anti-myeloperoxidase (MPO, 50 μg/ml, R&D) antibodies in blocking buffer overnight at 4 °C. The cells were stained for 2 h at room temperature with the corresponding Alexa 555 conjugate (1:200, Santa ctuz) and Alexa 488 conjugate secondary antibodies (1:500, Abcam). The coverslips were then mounted on glass slides using 4′,6-diamidino-2-phenylindole (DAPI) containing mounting medium (Beyotime). NET formation was counted as MPO and H3cit positive cells from at least three representative immunofluorescence images. NET formation was determined as the percentage of the field of H3cit-staining. Fluorescence values were obtained by Iamge J software.

Alternatively, the slides with neutrophils in the lower chamber were added with 1 μl cell-impermeable DNA dye SytoxGreen solution and 200 μl cell-permeable DNA dye Hoechst33342 solution (Biyuntian, Shanghai). After incubation for 20 min, immunofluorescence images were taken from at least three different views.

### Animal studies

Lipopolysaccharide (LPS, Sigma, 10 μg/mouse) was intraperitoneally injected to induce systemic inflammation in BALB/c mice. Six hours later, 2 × 10^6^ 4T1 cells were injected through the tail vein of each mouse. Mice were randomized on day five, and 100U/mouse DNase I (Roche), 15 mg/kg simvastatin or 0.9% normal saline was given intraperitoneally and 40 mg/kg BBR was given through oral administration daily until dissection. In vivo bioluminescence images were taken every 3 days using the IVIS Spectrum imaging system (PerkinElmer) to monitor tumor growth. Bleeding from the retro-orbital venous plexus of mice before dissection and separated serum. Serum were collected for MPO-DNA level detection. The mice were euthanized 25 days after injection of cancer cells. H&E stainings on paraffin-embedded lung tissues were used to calculate the metastatic area. Five mouse samples were randomly selected from each group and at least three discrete sections of each sample were analyzed.

### Immunohistochemical (IHC) staining and immunofluorescence (IF) staining

IHC staining was performed on formalin-fixed and paraffin-embedded sections of lung metastases with antibodies of anti-HMGCR (1:100, Santa cruz), anti-CCDC25 (1:100, Cell Signaling Technology), anti-MPO (1:200, R&D), anti-H3cit (1:300, Cell Signaling Technology), or anti-Caveolin-1 antibody (1:100, Cell Signaling Technology). Details can be found in the supplementary data.

Freshly obtained mouse lung tissues were quick-frozen in liquid nitrogen and placed in a cryostat to prepare frozen sections, each with a thickness of 4 mm. For IF staining, slides were rewarm at room temperature for 30 min and then combined with antibodies against CCDC25, Caveolin-1, MPO and H3cit overnight at 4 °C. Then rinsed the sections with ice PBS, and combined with secondary antibodies, respectively. Added DAPI-containing mounting medium, followed by mounting with a coverslip.

### Patient samples

Tissue microarrays (HBre-Duc060Cs-01) were constructed by Shanghai OutDo Biotech Co., LTD with cord No. 2005DKA21300. Sixty samples of human breast cancer course for microarray were obtained from patients with different degrees of breast lesions between 2004 and 2009. The clinicopathological characteristics of the patients are summarized in Supplementary Table 1.

### Statistical analysis

All data we use software for statistical analysis GraphPad Prism 7.0 (Graphpad Software, Inc.). All experiments were repeated at least three times. Data were represented as mean ± SD in the figures. *P*-values were calculated using the Student’s paired t-test. Statistically significant differences comparing three or more groups were analyzed using one-way or two-way analysis of variance (ANOVA) followed by the Bonferroni post-hoc test. Analysis of protein correlations using Fisher's test. Kaplan–Meier analysis was used to achieve survival analyses of patients with breast cancer in The Cancer Genome Atlas (TCGA) (n = 950). Value < the median of RNAseq gene expression were considered as low; value ≥ the median of that were considered as high. Difference in survival between groups was evaluated by the log-rank test. Differences were considered statistically significant at *P < 0.05, **P < 0.01, ***P < 0.001.

### Ethical approval

All animal experiments were performed in compliance with the ARRIVE guidelines. The study was approved by the Animal Ethics Committee of Shanghai University of Medicine and Health Sciences. The Ethics code is 2022-SZR-18-45010319800509104X. All methods were carried out in accordance with relevant guidelines and regulations.

### Animal sources

Female Balb/C mice, 6–8 week of age, 20–25 g weight, were purchased from Shanghai Jihui Laboratory Animal Care Co.,Ltd. (Shanghai, China) and were housed under specifc pathogen-free (SPF) conditions at the Shanghai University of Medicine & Health Science Animal Resource Center.

### Cell line authentication

Mouse breast cancer cell 4T1 and human breast cancer cell MDA-MB-231 were obtained from Shanghai Institute of Biotechnology, Chinese Academy of Science. The cell lines were expanded and cryopreserved according to ATCC guidelines, and regularly checked for mycobacteria.

## Results

### Cholesterol biosynthesis enhances tumor initiation abilities

**Figure 1:**
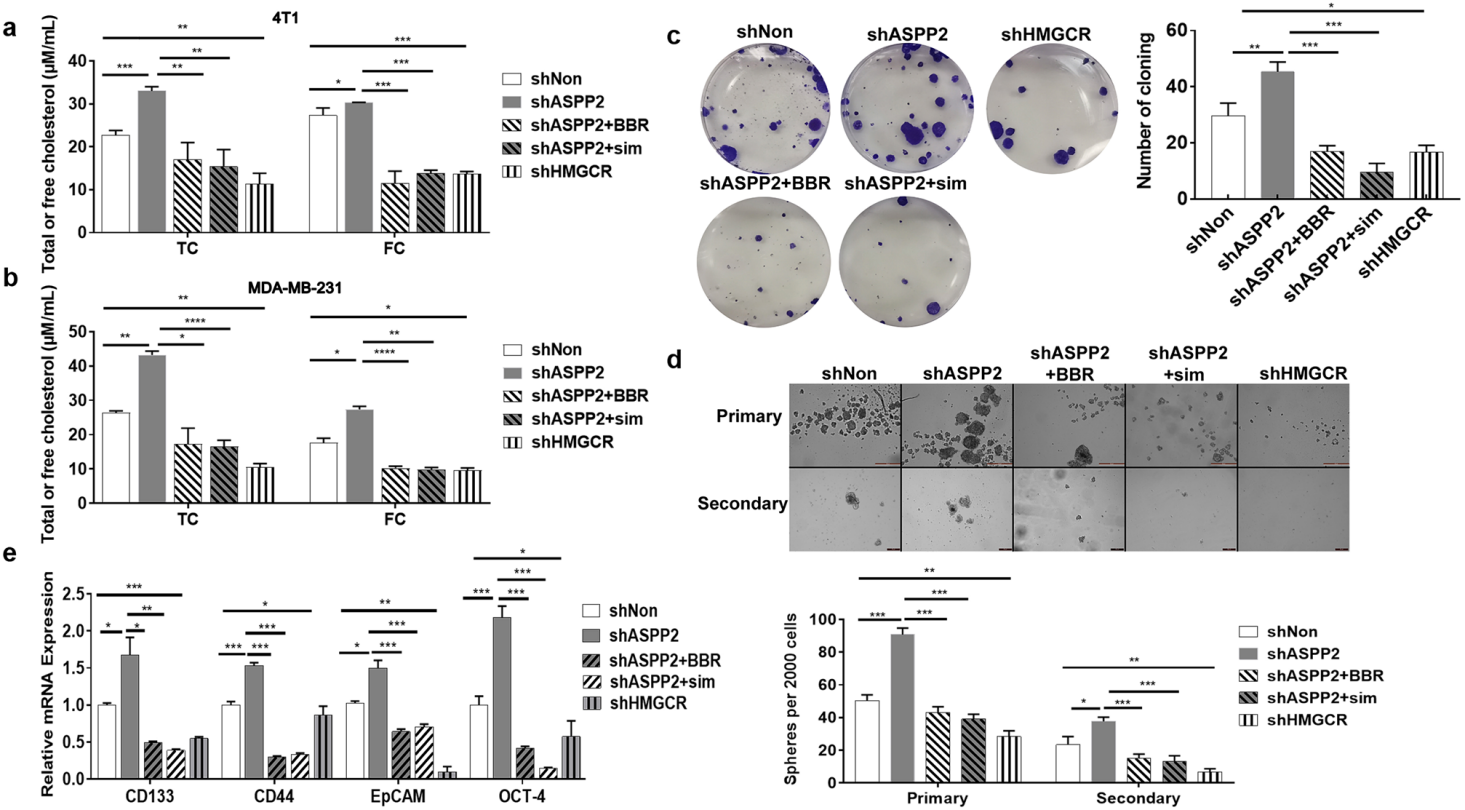
Cholesterol biosynthesis enhances colony and sphere formation in breast cancer cells. 4T1 (**a**) and MDA-MB-231 (**b**) cells were infected with lentivirus expressing shRNA targeting ASPP2 or HMGCR, and scramble shRNA (shNon) as control. 2 μM Simvastatin or 10 μM BBR were added to LV-shASPP2 infected cells. 24 h later, cells were collected and used for cholesterol content assays. (**c**) 2 × 10^3^ 4T1 cells per well were cultured in 6-well plates for 24 h, then cultured in medium containing 20% FBS for 12-15d. Colony number and representative images (left) were taken. (**d**) 1 × 10^5^ 4T1 cells were cultured onto ultralow attachment plates. Number of sphere formation in primary (6d) and secondary spheres (14d) were calculated. Representative images (left) was shown. Scale bar: 50 µm. (**e**) qRT-PCR assay for CD133, CD44, EpCAM and OCT-4 mRNA levels in treated 4T1 cells. *P < 0.05; **P < 0.01; ***P < 0.001.

We have previously reported that ASPP2, an activator of p53, negatively regulated MVA pathway by repressing SREBP2 transcription activity and the expressions of its target genes like HMGCR and HMGCS1^31^. We used shRNA-mediated gene knockdown to deplete ASPP2 or HMGCR expression in breast cancer cells MDA-MB-231 and 4T1 to generate cancer cells with enhanced or decreased cholesterol biosynthesis. Free and total cholesterol level, *HMGCR* mRNA, were significantly increased by ASPP2 depletion in cancer cells (Figs. 1a,b, and S2), which could be blocked by simvastatin and BBR, a new cholesterol-lowering drug that enhances LDLR expression or increases phosphorylation of HMGCR^35,36^. Cells expressing scramble shRNA (shNon) or shHMGCR were used as controls. To elucidate the effects of cholesterol biosynthesis on tumor initiation ability, colony formation in 6-well plates and sphere formation in a suspension culture system were assayed. The number of clones in shASPP2 4T1 cells were significantly higher than that in shNon cells. In contrast, the number of clones in shHMGCR 4T1 cells were significantly lower than that in control cells (Fig. 1c). Similar results were obtained in sphere formation assay. The number and the size of primary spheres and secondary spheres were greatly increased in 4T1 cells with ASPP2 depletion, and decreased in 4T1 cells with HMGCR depletion compared to control cells (Fig. 1d). Moreover, the expressions of putative stemness-associated markers CD44, CD133, EpCAM and transcription factor Oct4 were increased in shASPP2 cells and decreased in shHMGCR cells compared to shNon cells (Fig. 1e). Both simvastatin and BBR efficiently attenuated the effects induced by ASPP2 depletion (Fig. 1c–e). These data suggest that enhanced cholesterol biosynthesis endows tumor cells with higher tumor initiation ability, which benefits tumor development and progression.

### Cholesterol biosynthesis promotes invasion and formation of NETs in vitro

**Figure 2:**
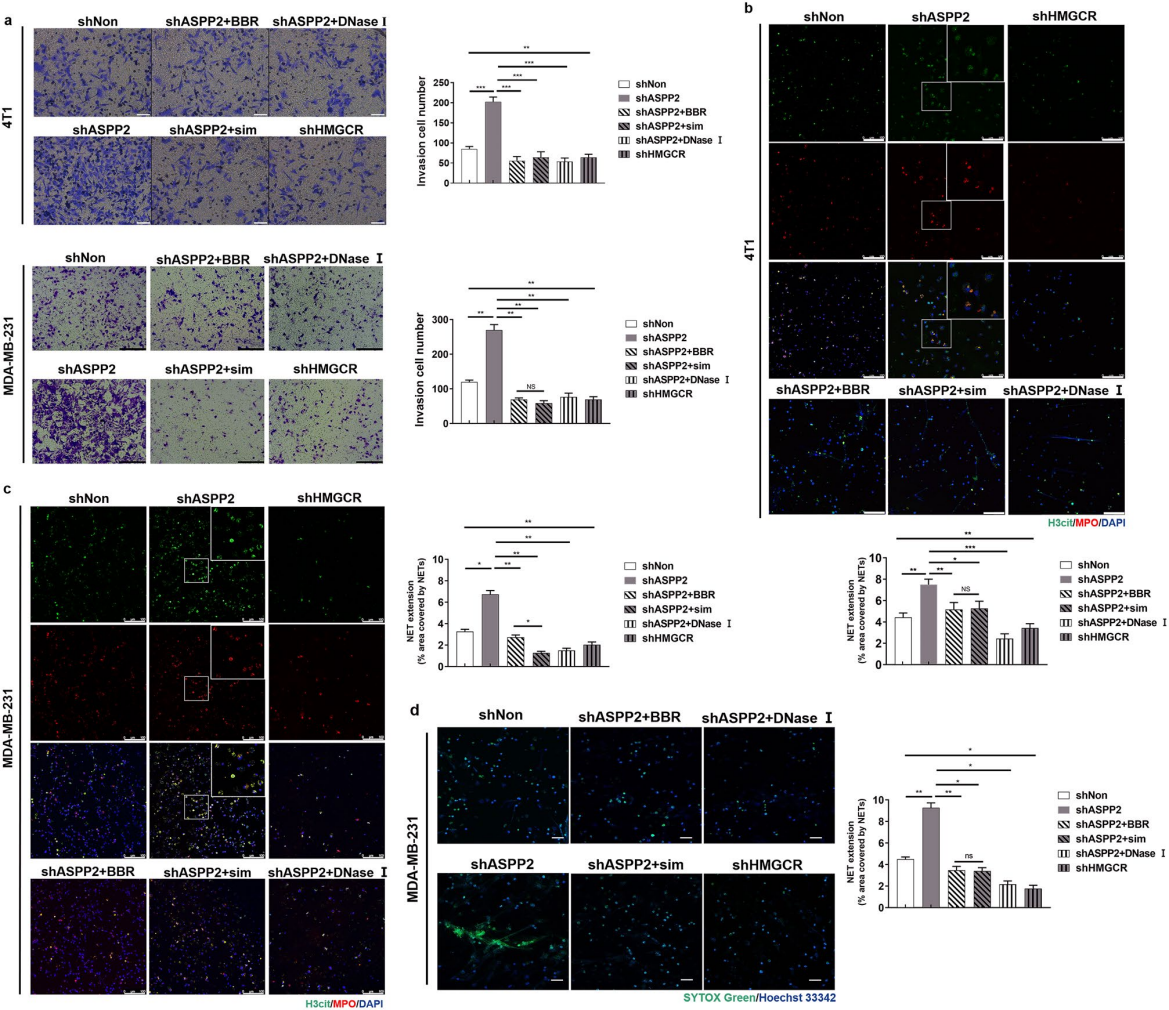
Cholesterol biosynthesis promotes cancer cells invasive abilities and formation of NETs in vitro. (**a**) 4T1 and MDA-MB-231 cells cultured with Matrigel in the upper chamber of the Transwells. 10 μM BBR, 2 μM simvastatin or DMSO was added to shASPP2-infected cancer cells. PMA stimulate neutrophil in serum-free medium with or without DNase I were add to the bottom wells. After cultured 22–26 h, counted invading cells by a light Microscope. Invasion was calculated by compared with invading cells in shNon group. Scale bar: 200 μm. (**b,c**) NETs formation as detected by immunofluorescence staining for H3cit and MPO were analyzed on neutrophils-cultured slides with 4T1 cells (**b**) or MDA-MB-231 cells (**c**). The areas marked by the white boxes are shown magnified in the insets in the top right. Scale bar: 50 μm. (**d**) SytoxGreen stainings were performed on neutrophils-cultured slides with MDA-MB-231 cells. Scale bar: 50 μm. *P < 0.05; **P < 0.01;***P < 0.001.

Cholesterol is recognized as a risk factor for breast cancer and high cholesterol is associated with breast cancer metastasis^2,15^. 4T1 cells with enhanced cholesterol biosynthesis by ASPP2 depletion exhibited stronger abilities of migration and invasion induced by FBS than control cells, which were markedly attenuated by administration of simvastatin and BBR (Fig. S3a and S3b). Whereas, 4T1 cells with reduced cholesterol biosynthesis by HMGCR depletion showed impaired abilities of migration and invasion (Fig. S3a,b). Recent studies have demonstrated the metastatic cancer cells are more prone to induce NETs formation^23,24^, we therefore analyzed the impact of cholesterol biosynthesis on NETs formation. In a co-culture system, neutrophils isolated from the mouse bone marrow were stimulated with PMA and plated in the lower wells, and cancer cells were put on matrigel in the upper wells. MDA-MB-231 and 4T1 cells with ASPP2 depletion exhibited more invasive cells than control cells, which could be suppressed by simvastatin and BBR (Fig. 2a). Whereas, cancer cells with HMGCR depletion showed fewer invasive cells than control cells (Fig. 2a). Thus, cholesterol biosynthesis promotes cancer cell invasion attracted by neutrophils. Interesting, administration of DNase I markedly suppressed invasion of ASPP2 depleted cancer cells, which indicates the involvement of NETs formation. To determine the extent of NETs, we performed IF staining with MPO, a marker of neutrophils and H3cit, a hallmark of chromatin decondensation. The extension of NETs were significantly elevated in ASPP2 depleted cells and reduced in HMGCR depleted cells as compared with control cells (Fig. 2b,c). ASPP2-depletion induced NETs formation were dramatically attenuated by simvastatin and BBR (Fig. 2b,c), which were partially compromised by supplementation of MVA pathway metabolites mevalonic acid 5-phosphate (MVAPP) (Fig. S4). Staining with cell-impermeable DNA dye SytoxGreen also showed stronger NETs formation in ASPP2 depleted MDA-MB-231 cells than in control cells (Fig. 2d), which could be blocked by simvastatin, BBR and DNase I. These data suggest that cholesterol biosynthesis promotes invasive ability of cancer cells and the ability to form NETs with neutrophils.

### Cholesterol biosynthesis increases lipid raft-mediated CCDC25 expression

**Figure 3:**
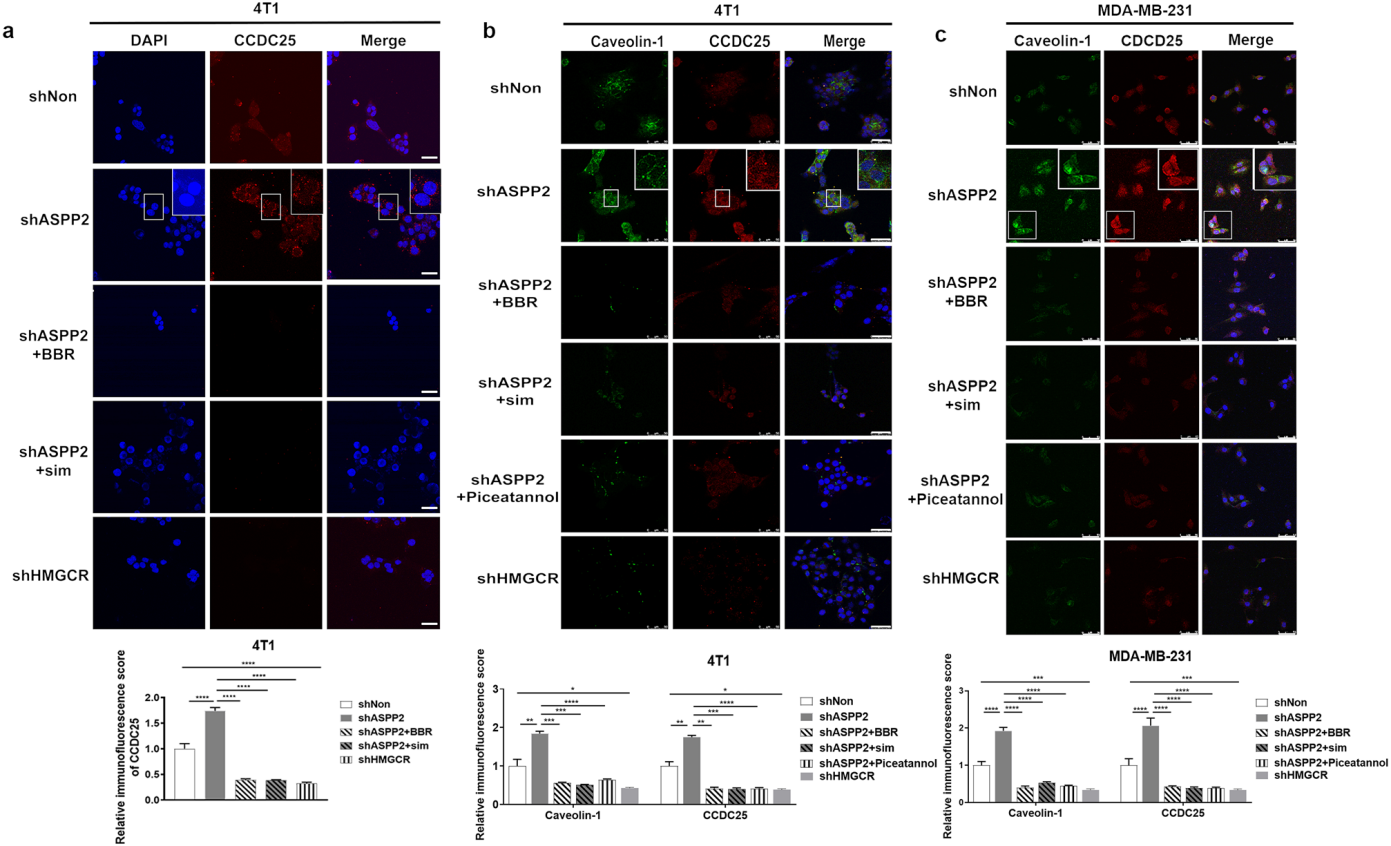
CCDC25 expressing is mediated by lipid rafts. (**a**) 4T1 cells as indicated were subjected to immunofluorescence staining of CCDC25. DAPI (blue stain) was used to stain the nucleus. Scale bars: 50 μm. (**b,c**) Confocal microscopy images showing the co-localization of Caveolin-1 (green staining) with CCDC25 (red staining) in 4T1 (**b**) and MDA-MB-231 (**c**) cells. The areas marked by the white boxes are shown magnified in the insets in the top right. Piceatannol was used to inhibition lipid rafts formation. Scale bar: 50 µm. Relative immunofluorescence scores were calculated and statistically analyzed. *P < 0.05; ** *P* < 0.01;*** *P* < 0.001.

Previous study has identified the transmembrane protein CCDC25 on cancer cells as a specific sensor for NET-DNA^25^. Interestingly, ASPP2 depletion greatly strengthened the expression of CCDC25 on 4T1 cell membrane (Fig. 3a). Conversely, HMGCR depletion attenuated CCDC25 expression obviously (Fig. 3a). ASPP2-depletion induced CCDC25 expression was damped by inhibiting cholesterol biosynthesis with simvastatin and BBR (Fig. 3a). Lipid rafts are cholesterol and glycosphingolipid enriched membrane microdomains that act as platforms to promote transmembrane signaling^37^. We therefore examined whether CCDC25 expression was related with lipid rafts formation. Notably, ASPP2 depletion greatly increased the amount caveolin-1, a critical protein in lipid rafts, which was co-localized with CCDC25 on cell surface in 4T1 and MDA-MB-231 cells (Fig. 3b,c). Simvastatin and BBR lessened caveolin-1 and CCDC25 expression concurrently. Further, blocking lipid rafts polarization with piceatannol, a spleen tyrosine kinase inhibitor, greatly eliminated caveolin-1 and CCDC25 expression (Fig. 3b,c). Thus, cholesterol-induced CCDC25 expression is associated with lipid rafts. Our data reveal that enhanced cholesterol biosynthesis promotes lipid raft-mediated CCDC25 expression on cancer cell membrane to benefit the formation of NETs.

### Inhibition of cholesterol biosynthesis suppresses metastasis and NETs in vivo

**Figure 4:**
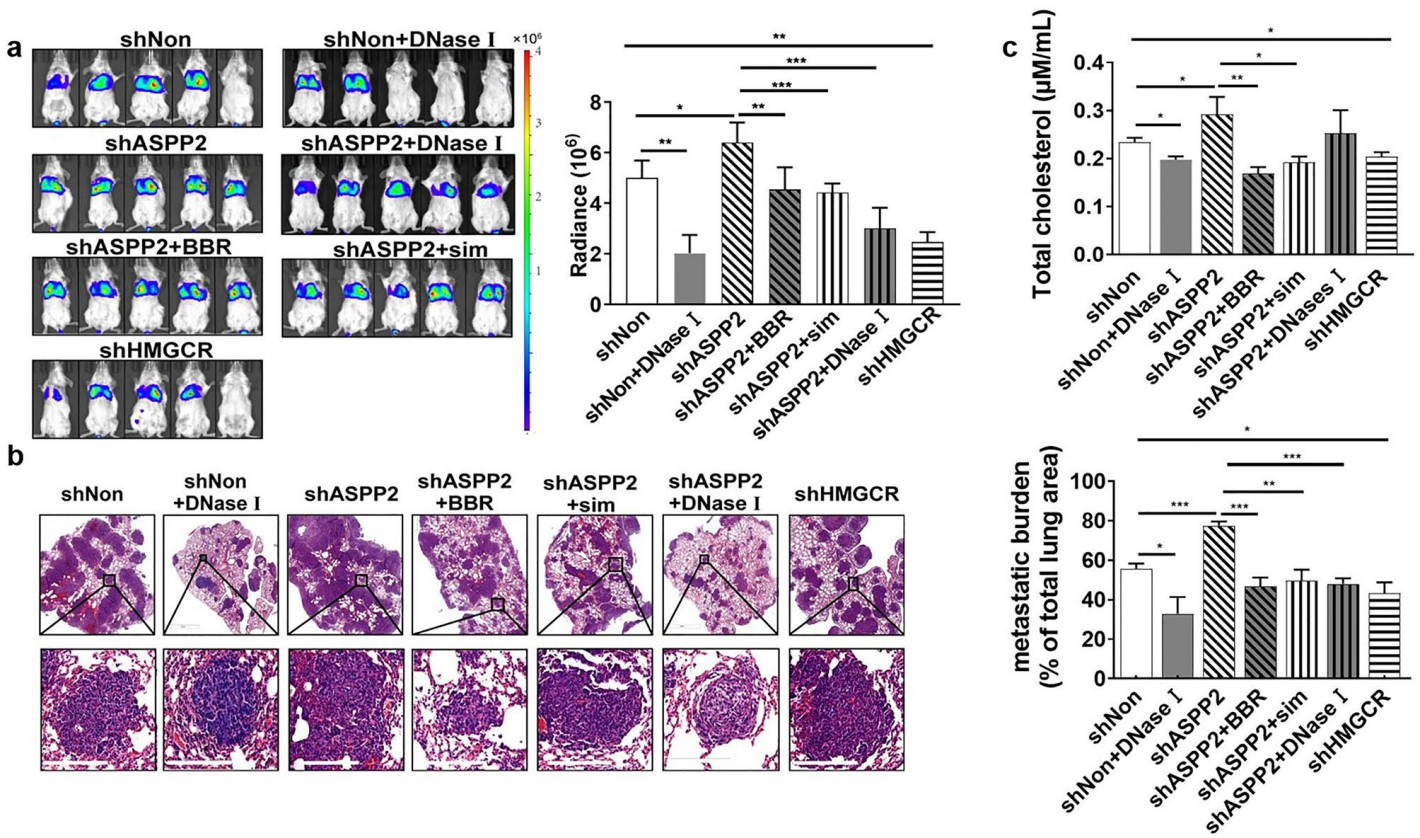
Inhibition of cholesterol biosynthesis suppresses lung metastasis of breast cancer in LPS-stimulated mice. (**a**) Female BALB/c mice were intraperitoneally injected with LPS for 6 h before luciferase expressing 4T1 (1 × 10^6^) cells were injected through the tail vein. Inducible shRNA-luc expressing 4T1 cells were monitored by BLI every 3 days. Simvastatin, BBR or DNase I treatment was initiated after randomization on day 5 (n = 5 mice per group; means ± SD). Representative BLI images at day 25. The densities of BLI images were calculated and statically analyzed. (**b**) Representative H&E images of the lung metastases in LPS-stimulated mouse model. (n = 10 each). Lung metastatic burdens were assessed by comparing metastatic area to total lung area. Scare bar: 200 µm. (**c**) Mice are bled from the retro-orbital venous plexus on day 24, and serums are collected. Cholesterol levels are determined and statically analyzed. *P < 0.05; **P < 0.01; ***P < 0.001.

To further verify the effects of cholesterol metabolism on breast cancer metastasis and NETs in vivo, 4T1 cells expressing shASPP22-Luc, shHMGCR-Luc or shNon-Luc were injected through the tail vein into mice pretreated with LPS intraperitoneally. Twenty-five days after inoculation, bioluminescence imaging showed the signal intensity of lung metastases in shASPP2 group was remarkably higher than that in shNon group, which could be inhibited by the treatment of cholesterol biosynthesis inhibitors or DNase I (Fig. 4a). The percentage of metastatic burden was much higher in ASPP2-depletion group than control group, which was significantly reduced by the treatment of cholesterol biosynthesis inhibitors or DNase I (Fig. 4b). Subsequently, we measure the serum cholesterol level in mice, which showed enhanced cholesterol level upon ASPP2 depletion (Fig. 4c).

**Figure 5:**
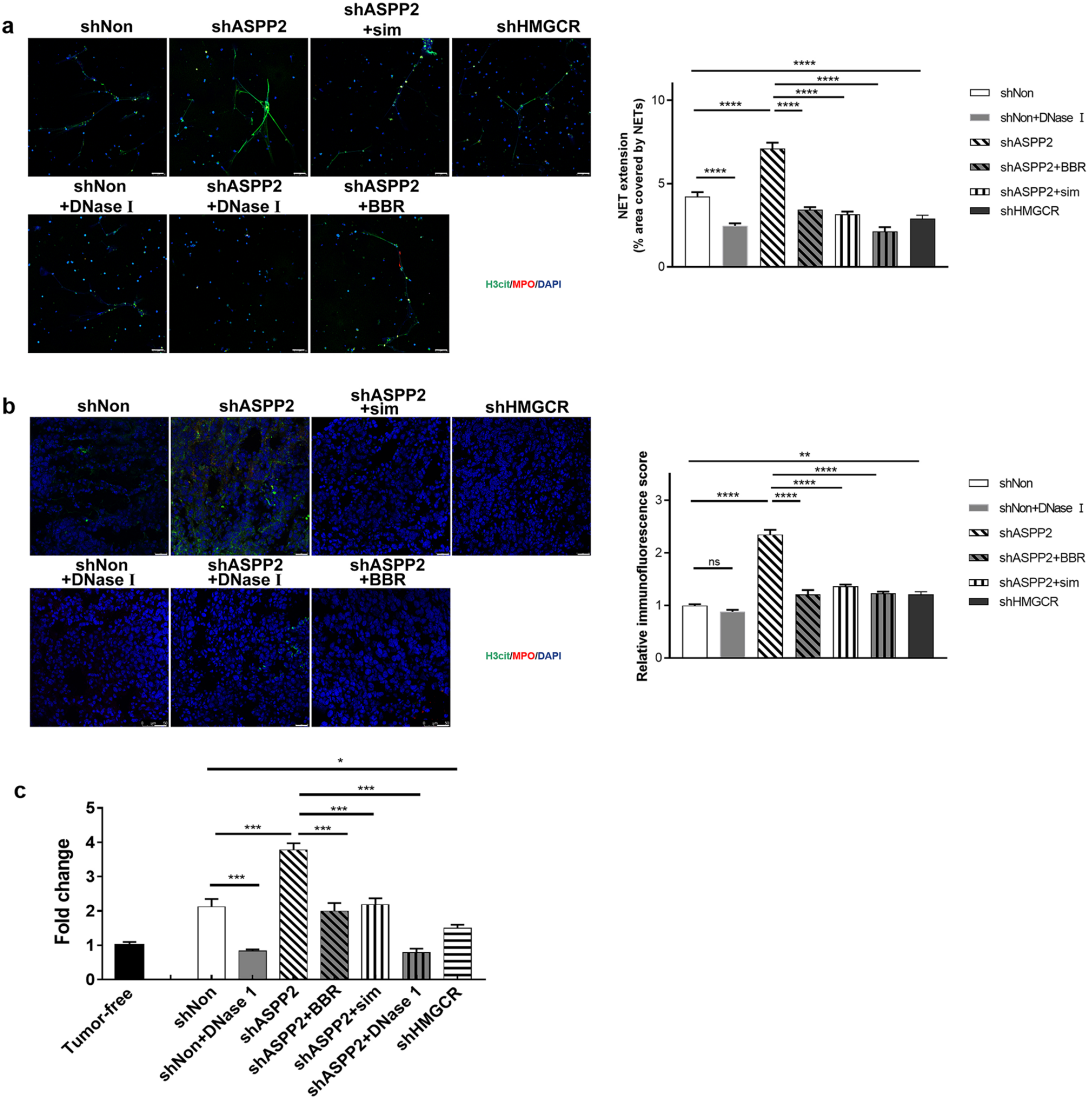
Inhibition of cholesterol biosynthesis attenuates NETs formation in lung metastases. (**a**) Images showed immunostaining of neutrophils cultured on poly-L-lysine-coated coverslips in 6 well plant. Neutrophils were isolated from femur and tibia of indicated mice as described in Fig. 4. MPO (red), H3cit (green) and DAPI (blue) staining were used to assess NETs formation. Scale bar: 50 µm. (**b**) Images showed representative immunostaining for MPO, H3cit and DAPI in lung metastases of indicated mice. Scale bar: 50 µm. (**c**) MPO-DNA levels in serum samples from indicated mice before dissection (n = 5 each). 0.9% saline served as a control. Data were normalized to healthy mouse serum. *P < 0.05; ***P < 0.001.

To verify the NETs formation in different groups, we isolated neutrophils from mouse femur and tibia. The image showed that neutrophils from ASPP2-depletion mice exhibited stronger NETs structure than that from control mice, conversely HMGCR depletion or treatment with cholesterol inhibitors weakened NETs formation (Fig. 5a). Further, increased neutrophil infiltration and NETs in lung metastases were observed in ASPP2-depletion mice as shown by IF staining of neutrophil and NETs markers (Fig. 5b). NETs formations were greatly reduced by the treatment of cholesterol biosynthesis inhibitors or DNase I in ASPP2 deficient mice (Fig. 5a,b). We then detected serum MPO-DNA level, a NET product to confirm NETs formation, which showed the consistent results with previous observations (Fig. 5c).

**Figure 6:**
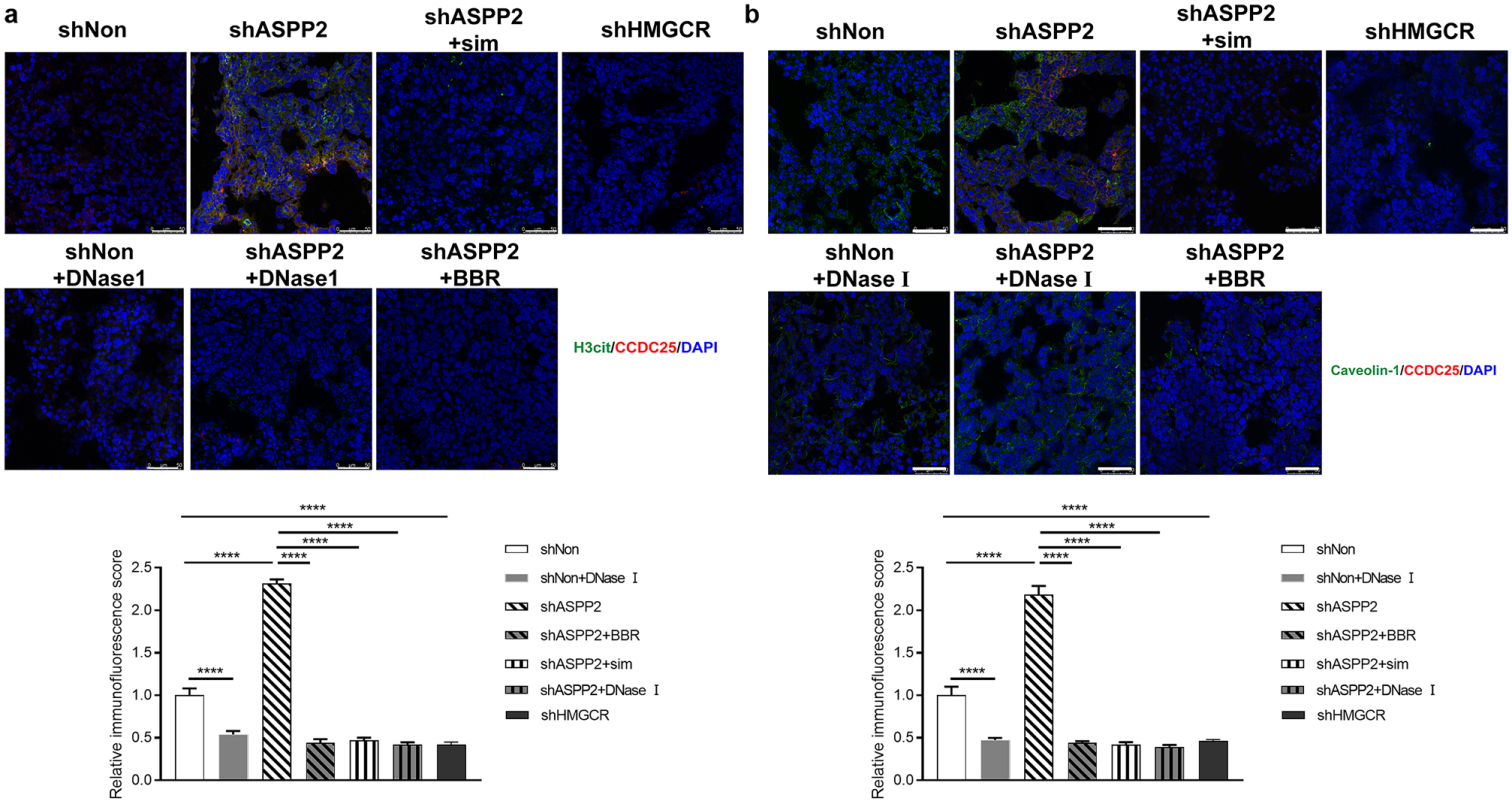
Inhibition of cholesterol biosynthesis reduces CCDC25 expression in lung metastases. (**a**) Images showed representative immunostaining for CCDC25 (red), H3cit (green) and DAPI (blue) in the lung metastases of indicated mice as in Fig. 4. Scale bar: 50 µm. (**b**) Images showed representative immunostaining for CCDC25, Caveolin-1 and DAPI in the lung metastases of indicated mice. Scale bar: 50 µm. Relative immunofluorescence scores were calculated and statistically analyzed. *P < 0.05; ** *P* < 0.01;*** *P* < 0.001.

In consistence with previous in vitro findings, CCDC25 expression was greatly enhanced in lung metastases from ASPP2-depletion mice, in coordination with increased NETs formation, as detected by IF staining (Fig. 6a). CCDC25 expression was greatly alleviated by the treatment of cholesterol biosynthesis inhibitors or DNase I (Fig. 6a). Moreover, lipid raft was increased coordinately with CCDC25 boost, with caveolin-1 co-localized with CCDC25 in lung metastases, which was damped by the treatment of cholesterol biosynthesis inhibitors (Fig. 6b).

**Figure 7:**
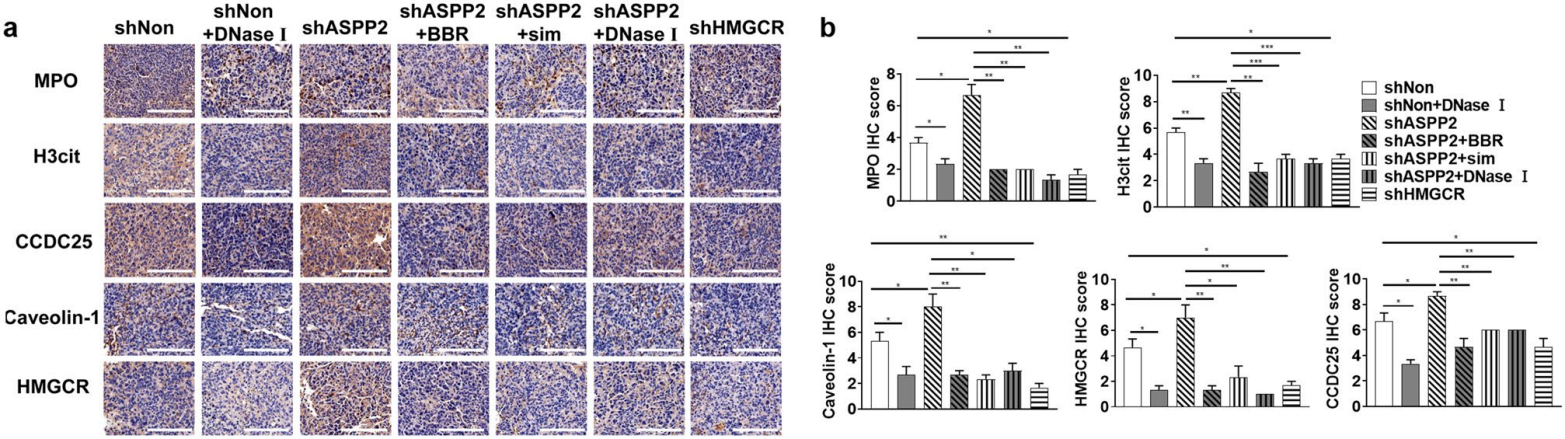
Immunohistochemical staining in lung metastases. (**a**) Representative images of IHC assays for H3cit, MPO, Caveolin-1, CCDC25 and HMGCR of lung tissues from indicated mice as in Fig. 4. Scale bar: 200 µm. (**b**) Statistical analysis of IHC scores. *P < 0.05; **P < 0.01; ***P < 0.001. Data were presented as means ± SD.

IHC staining further confirmed previous findings. ASPP2 depletion caused HMGCR up-regulation in lung metastases, recruited more neutrophils as detected by MPO staining and induced stronger NETs formation as indicated by H3cit staining (Fig. 7a,b). ASPP2-deficient induced cholesterol biosynthesis also increased lipid rafts formation detected by caveolin-1, concurrently with enhanced CCDC25 expression (Fig. 7a,b). Treatment of DNase I not only blocked NETs formation but also decreased CCDC25 expression in metastases, which indicates that inhibition of NETs formation disturbs the process by which cancer cells be attracted to the metastatic sites.

Therefore, cholesterol-induced tumor metastasis was associated with enhanced CCDC25 expression on cancer cells and NETs formation in metastatic niches in mouse breast cancer model. Inhibiting cholesterol biosynthesis efficiently suppresses NETs formation and metastasis in vivo*.*

### Cholesterol synthesis is associated with enhanced CCDC25 expression in breast cancer patients

**Figure 8:**
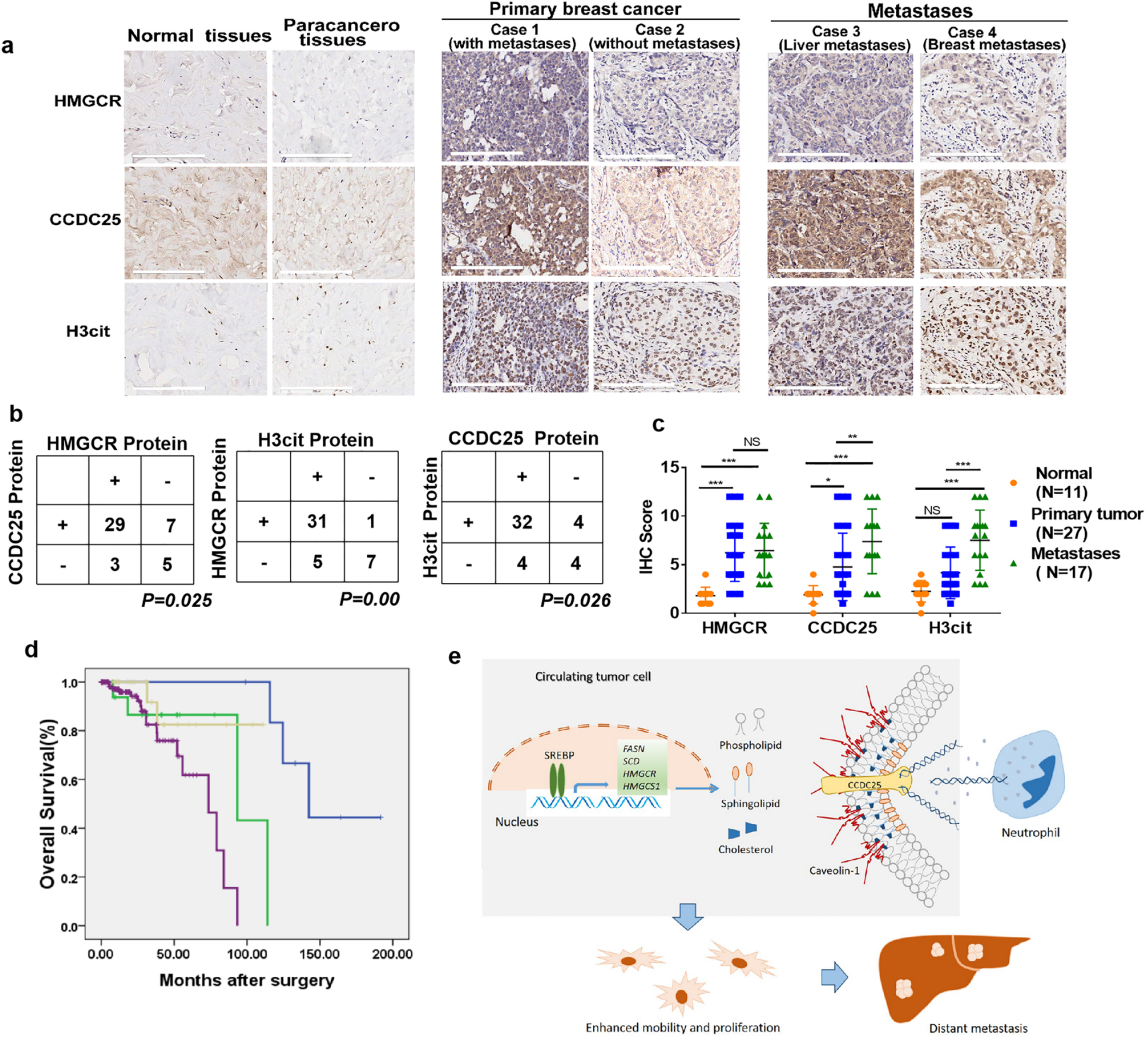
Tissue microarray staining and statistical analysis. (**a**) CCDC25, H3cit and HMGCR were detected by immunohistochemical staining in a tissue microarray containing 60 breast tissues. Representative pictures of IHC staining in normal or papacancerous breast tissues, primary breast cancer tissues, and metastatic tissues. Scale bars: 200 µm. (**b**) Statistical analyses of the correlations between HMGCR and CCDC25, HMGCR and H3cit, CCDC25 and H3cit, according to immunohistochemical scoring by Fisher's test. (**c**) Statistical analyses IHC score of HMGCR, CCDC25 and H3cit in normal tissues, primary breast cancer tissues and metastatic tissues by Two-way ANOVA test. (**d**) Kaplan–Meier curves showing the overall survival of patients with breast cancer with high or low CCDC25 and HMGCR expression in The Cancer Genome Atlas (TCGA) breast cancer online database (n = 176). Comparisons were performed using a log rank test. *P* < 0.05; **P < 0.01; ***P < 0.001. (**e**) The schematic model depicts how cholesterol biosynthesis contributes to CCDC25 expression, NETs formation and distant metastases.

To assess the clinical impact of cholesterol biosynthesis on breast cancer metastasis and NETs, we examined HMGCR, CCDC25 and H3cit expression by IHC staining in a tissue array, including 11 normal and paracancerous breast tissues, 27 primary breast cancer tissues and 17 metastatic tissues (Fig. 8a,b). The clinical pathological parameters of patients were shown in Supplementary Table1. HMGCR, CCDC25 and H3cit expressions were negative [score value: ≤ 2 (range 0–2)] in normal and paracancerous tissues, and positive in primary breast cancer tissues and metastatic tissues [score value: > 2 (range 2–12)] (Fig. 8a). CCDC25 was detected on cell membrane and in cytoplasmid, and was found high expressed [score value:12 (range, 2–12] in a primary cancer tissue from patient with ovary metastasis and in a liver metastatic tissue (Fig. 8a, case 1 and case 3). The relationships between HMGCR, CCDC25, H3cit expression and clinical features in 27 primary breast cancer patients were then statistically analyzed, which exhibited no significant correlations probably due to the limitation of tissue amount (Supplementary Table2). However, the expression of HMGCR was positively correlated with the expression of CCDC25 and H3cit in 44 primary and metastatic cancer tissues (*P* < 0.05; Fig. 8b), and the expression of CCDC25 and H3cit was also positively correlated (*P* < 0.05; Fig. 8b). Further, the average IHC scores of CCDC25 and H3cit was significantly higher in metastatic tissues than that in primary cancer tissues (*P* < 0.05; Fig. 8c). Patients with breast cancer (n = 176) in The Cancer Genome Atlas (TCGA) breast cancer online database were divided into four subgroups HMGCR^low^/CCDC25^low^ (n = 7), HMGCR^low^/CCDC25^high^ (n = 17), HMGCR^high^/CCDC25^low^ (n = 25), HMGCR^high^/CCDC25^high^ (n = 127). Kaplan–Meier analysis showed overall survival were significantly worse among patients with HMGCR^high^/CCDC25^high^ (*P* < 0.01, Fig. 8d). These data suggest that cholesterol biosynthesis is a positive regulator for the expression of CCDC25 in cancer cells and NETs formation. Clinically, enhanced CCDC25 and HMGCR expressions are related with worse prognosis in breast cancer patients. The schematic model depicting how cholesterol biosynthesis contributes to CCDC25 expression, NETs formation and distant metastases is shown (Fig. 8e).

## Discussion

Enhanced cholesterol biosynthesis has been linked closely with stem-like characteristics in breast cancer, which are important for tumor initiation and metastasis. MVA pathway enhanced by mutant p53 was essential for breast cancer cell to maintain morphology in 3D culture^29^. Gene expression analysis revealed the enzymes of the MVA pathway were overexpressed significantly in basal/mesenchymal tumor spheres compared to adherent cancer cells^17^. Mammospheres generated from breast cancer tissues exhibited increased expression of proteins associated with cholesterol synthesis^18^. Compared with human primary breast tumors and lung metastases, the matched lung metastases displayed markedly elevated levels of HMGCR and HMGCS1^38^. The clinical relevance of increased cholesterol biosynthesis was verified in breast cancer cohort showing correlation with poorer survival probability^18,29^. Here we used lentivirus mediated shRNA expression to deplete ASPP2 expression and generated 4T1 mouse breast cancer cells with enhanced cholesterol biosynthesis. ASPP2 depletion increased HMGCR expression, endowed 4T1 cells with enhanced abilities to form spheres, migration and invasion stimulated by FBS and neutrophils.

NETs extruded by neutrophils have drawn a lot of attention in the regulation of breast cancer metastasis^23–25^. Regulation of NETs formation in lung metastasis of breast cancer has recently been reported by tumor-secreted proteins Cathepsin C or lung mesenchymal stromal cells produced complement 3^39,40^. Though it has been shown that cholesterol metabolite 27-hydroxycholesterol promoted the recruitment of neutrophils to metastatic site^15^, the impact of de novo cholesterol biosynthesis on NETs formation remains unclear. Previous work has shown that compared to non metastatic 4T07 cells, metastatic 4T1 cells were capable to induce NETs formation^23^. The underline mechanism for this difference has not been answered. Therefore, we determine to explore whether de novo cholesterol biosynthesis in cancer cells makes the difference of NETs formation with neutrophils.

Here we found ASPP2-deficient induced cholesterol biosynthesis in 4T1 cells greatly enhanced NETs formation when co-cultured with neutrophils in vitro*.* Neutrophils isolated form mice intravenously injected with ASPP2 deficient 4T1 formed extensive NETs. Intravenous injection of ASPP2 deficient 4T1 cells in LPS-stimulated mice aroused more neutrophils recruitment and stronger NETs structure. ASPP2 deficient induced NETs formation was efficiently blocked by cholesterol inhibitors simvastatin and BBR. These observations demonstrate the role of de novo cholesterol biosynthesis in neutrophils recruitment and NETs formation.

CCDC25 was identified as the transmembrane receptor of NET-DNA on cell surface to promote cell migration^25^. Overexpression of CCDC25 has been reported in breast and colon cancers collected in the Human Protein Atlas database^25^. The regulation of CCDC25 is largely unknown. Here, we demonstrated that cholesterol promoted CCDC25 expression in a lipid raft-dependent manner. Lipid rafts are tightly packed, cholesterol- and sphingolipid-enriched microdomains^37^. Lipid rafts have been implicated as important regulators of signal transduction during tumor progression by modulating tumor angiogenesis, cell adhesion, migration, and epithelial-mesenchymal transition^41^. Expression of malignancy related genes, like mucin 1, urokinase plasminogen activator surface receptor, and RAS, has been found in breast cancer in a raft-dependent manner^42–44^. Here, we found CCDC25 and caveolin-1 were increased and co-localized in ASPP2 deficient 4T1 cells, as well as in lung metastases from mice intravenously injected with 4T1 cells. Cholesterol inhibitors attenuated CCDC25 and caveolin-1 expression simultaneously. Lipid rafts inhibitor piceatannol greatly lessened CCDC25 expression. Together, our data demonstrate, for the time, regulation of CCDC25 by lipid rafts. The mechanism by which lipid rafts regulates CCDC25 expression needs further investigation.

Clinically, we detected HMGCR, CCDC25 and H3cit expression in a breast tissue array. Though no statistical relationships between HMGCR, CCDC25, H3cit expression and clinical features were found in 27 primary breast cancer patients, probably due to the limitation of tissue amount, the expression of CCDC25 was positively correlated with the expression of HMGCR and H3cit in 44 primary and metastatic cancer tissues, and the average IHC scores of CCDC25 was significantly higher in metastatic tissues than that in primary cancer tissues. These data suggest that cholesterol biosynthesis is a positive regulator for the expression of CCDC25, enhanced CCDC25 expression is related with breast cancer metastasis.

In conclusion, our study uncovers a novel mechanism for cholesterol biosynthesis in the regulation of NETs formation and breast cancer metastasis. Targeting cholesterol biosynthesis in tumor cells may be promising therapeutic strategies to treat metastasis or prevent recurrence.

## Supplementary Information

Supplementary Information.

## Data Availability

All remaining data are available within the article and supplementary files, or available from the authors upon request.

## Abbreviations

BBR: Berberine
CCDC25: Coiled-coil domain containing protein 25
DAPI: 4′,6-Diamidino-2-phenylindole
FBS: Fetal bovine serum
H3cit: Citrullinated histone H3
HMRCG: 3-Hydroxy-3-methylglutaryl-CoAreductase
IHC: Immunohistochemical
IF: Immunofluorescence
LPS: Lipopolysaccharide
MPO: Myeloperoxidase
MVA: Mevalonate
MVAPP: Mevalonic acid 5-phosphate
NETs: Neutrophil extracellular traps
PFA: Paraformaldehyde
PMA: Phorbol 12-myristate 13-acetate
SREBP-2: Sterol regulated element binding protein-2
