# Author Correction: Enhanced CHOLESTEROL biosynthesis promotes breast cancer metastasis via modulating CCDC25 expression and neutrophil extracellular traps formation

**DOI:** 10.1038/s41598-025-16659-1

**Published:** 2025-09-08

**Authors:** Qiqi Tang, Beibei Liang, Lisha Zhang, Xuhui Li, Hengyu Li, Wei Jing, Yingjie Jiang, Felix Zhou, Jian Zhang, Yanchun Meng, Xinhua Yang, Hao Yang, Gang Huang, Jian Zhao

**Affiliations:** 1 Shanghai Key Laboratory of Molecular Imaging, Shanghai University of Medicine and Health Science, 279Th Zhouzhu Road, Shanghai, 201318 China; 2 Shanghai Key Laboratory of Molecular Imaging, Jiading District Central Hospital Affiliated Shanghai University of Medicine and Health Sciences, Shanghai, 201318 China; 3 Shanghai University of Traditional Medicine, Shanghai, 201203 China; 4 Changhai Hospital, Navy Military Medical University, Shanghai, 200438 China; 5 Ludwig Institute for Cancer Research, Nuffield Department of Clinical Medicine, University of Oxford, Oxford, OX3 7DQ UK; 6 Phase I Clinical Trial Center, Shanghai Cancer Center, Fudan University, Shanghai, 200032 China; 7 Department of Oncology, Shanghai Medical College, Fudan University, Shanghai, 200032 China

Correction to: *Scientific Reports* 10.1038/s41598-022-22410-x, published online 17 October 2022

The original version of this Article contained errors.

As a result of an error during assembly of Figure 4A, two mice representing the shNon control were duplicated from the shASPP2+BBR condition. As a result, the corresponding bioluminescence statistical analysis was also incorrect. The original Figure 4 and accompanying legend appears below.

**Fig. 4:**
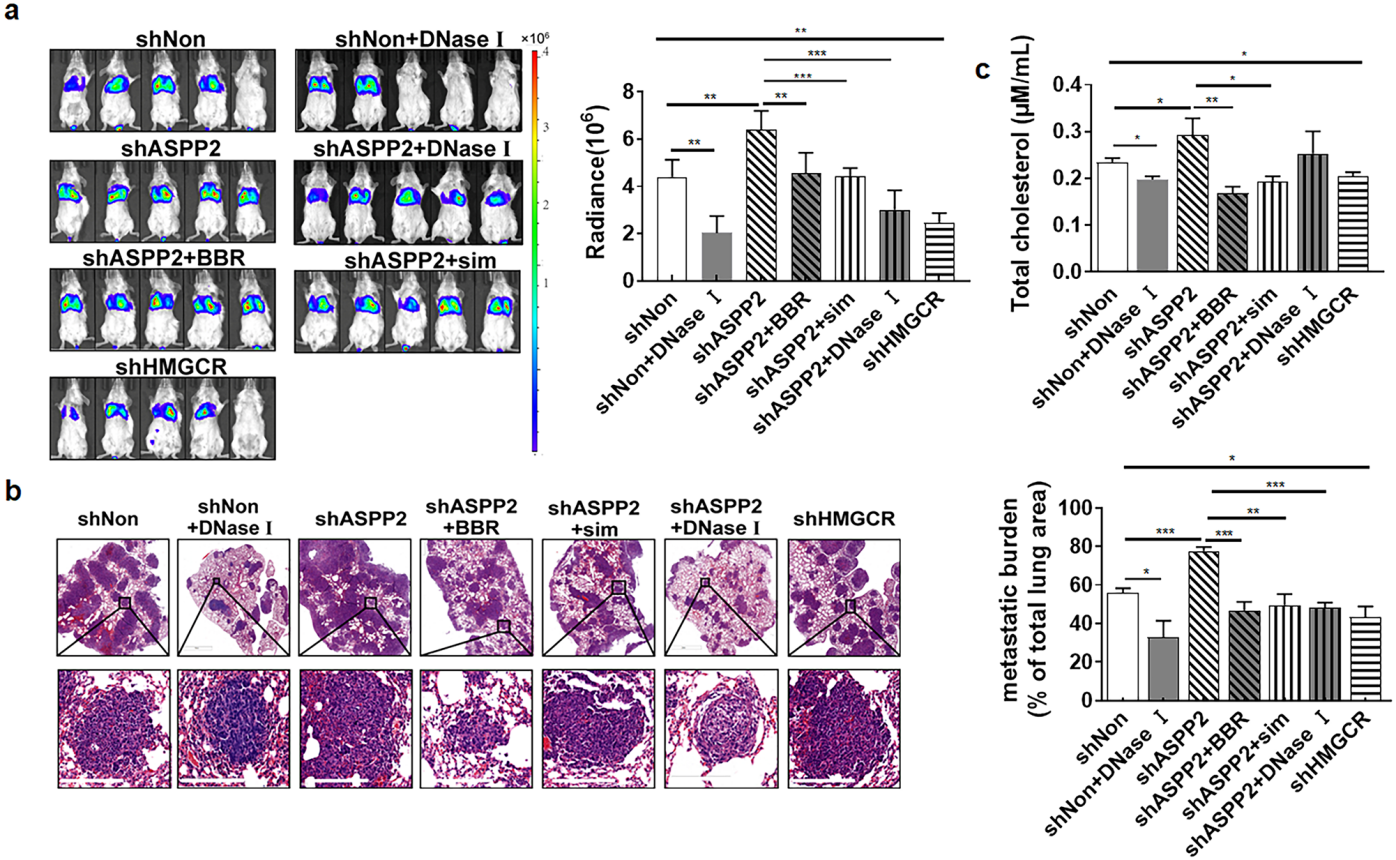
Inhibition of cholesterol biosynthesis suppresses lung metastasis of breast cancer in LPS-stimulated mice. (**a**) Female BALB/c mice were intraperitoneally injected with LPS for 6 h before luciferase expressing 4T1 (1 × 10^6^) cells were injected through the tail vein. Inducible shRNA-luc expressing 4T1 cells were monitored by BLI every 3 days. Simvastatin, BBR or DNase I treatment was initiated after randomization on day 5 (n = 5 mice per group; means ± SD). Representative BLI images at day 25. The densities of BLI images were calculated and statically analyzed. (**b**) Representative H&E images of the lung metastases in LPS-stimulated mouse model. (n = 10 each). Lung metastatic burdens were assessed by comparing metastatic area to total lung area. Scare bar: 200 µm. (**c**) Mice are bled from the retro-orbital venous plexus on day 24, and serums are collected. Cholesterol levels are determined and statically analyzed. *P < 0.05; **P < 0.01; ***P < 0.001.

In addition, as a result of an error during the assembly of Figure 8A, the CCDC25 immunohistochemical staining panel from Case 2 (without metastases) was duplicated from Case 4 (breast metastases). The original Figure 8 and accompanying legend appears below.

**Fig. 8:**
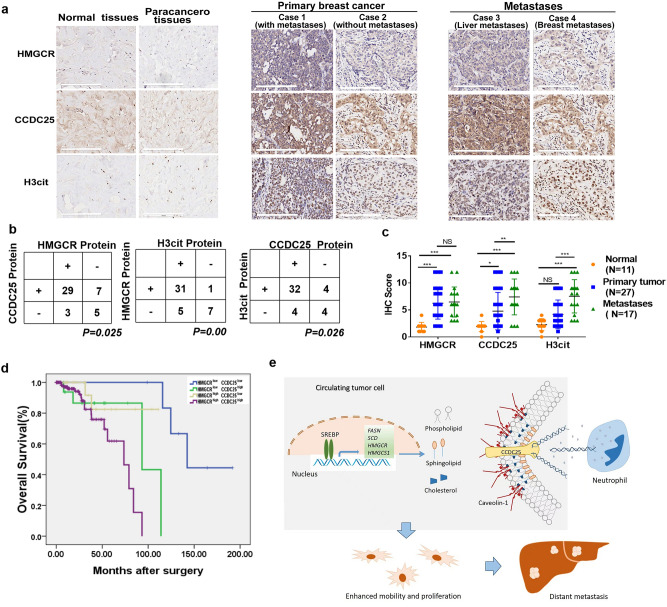
Tissue microarray staining and statistical analysis. (**a**) CCDC25, H3cit and HMGCR were detected by immunohistochemical staining in a tissue microarray containing 60 breast tissues. Representative pictures of IHC staining in normal or papacancerous breast tissues, primary breast cancer tissues, and metastatic tissues. Scale bars: 200 µm. (**b**) Statistical analyses of the correlations between HMGCR and CCDC25, HMGCR and H3cit, CCDC25 and H3cit, according to immunohistochemical scoring by Fisher’s test. (**c**) Statistical analyses IHC score of HMGCR, CCDC25 and H3cit in normal tissues, primary breast cancer tissues and metastatic tissues by Two-way ANOVA test. (**d**) Kaplan–Meier curves showing the overall survival of patients with breast cancer with high or low CCDC25 and HMGCR expression in The Cancer Genome Atlas (TCGA) breast cancer online database (n = 176). Comparisons were performed using a log rank test. *P* < 0.05; **P < 0.01; ***P < 0.001. (**e**) The schematic model depicts how cholesterol biosynthesis contributes to CCDC25 expression, NETs formation and distant metastases.

The original Article has been corrected.

