# Supplementary material for: Enhanced CHOLESTEROL biosynthesis promotes breast cancer metastasis via modulating CCDC25 expression and neutrophil extracellular traps formation
Source: Sci Rep. 2022 Oct 17;12:17350. doi: 10.1038/s41598-022-22410-x (PMC9576744; doi:10.1038/s41598-022-22410-x)
Supplement: Supplementary file 1 — Supplementary Information. [file 41598_2022_22410_MOESM1_ESM.pdf]

Supplementary Materials for

Enhanced Cholesterol biosynthesis promotes breast cancer metastasis *via* modulating CCDC25 expression and neutrophil extracellular traps formation

Qiqi Tang, Beibei Liang, Lisha Zhang, Xuhui Li, Hengyu Li, Wei Jing, Yingjie Jiang, Felix Zhou，Jian Zhang, Yanchun Meng, Xinhua Yang, Hao Yang, Gang Huang, Jian Zhao

Correspondence:

Jian Zhao, Shanghai Key Laboratory for Molecular Imaging, Shanghai University of Medicine & Health Science, 279th Zhouzhu Road, Shanghai 201318, P.R.China; e-mail: j-zhao@vip.126.com

Supplementary Materials and Methods

Quantitative real-time PCR (qRT-PCR)

Total RNA was extracted using RNeasy Mini kit (Qiagen) and reverse transcription was performed using a reverse transcription kit (Vazyme, Nanjing, China,). Complementary DNA (cDNA) was synthesized using HiScript II Q RT SuperMix (R223-01, Vazyme). Real-time PCR was completed using SYBR Green PCR Master Mix (Takara, Otsu, Japan) on QuantStudioTM 7 Flex (Thermo Fisher Scientific). All target genes were normalized to cyclophilin B. Gene expression was quantified using the delta Ct method. Primer sequences were as follows:

human HMGCR：forward, TGATTGACCTTTCCAGAGCAAG

reverse, CTAAAATTGCCATTCCACGAGC

human ASPP2 forward, AGATAGTGATGGATGGACGCC

reverse, CCATCTTCTCCTGAACGCA

mouse ASPP2 forward, 5′-AGATAGTGATGGATGGACGCC-3′

reverse, 5′-CTGGGTCATCTTTTCACGGT-3′

mouse HMGCR forward, 5′-CGATCCTTCCTTATTGGCGG-3′

reverse, 5′-CGGATCTCAATGGAGGCCAA-3′

mouse CD133 forward, 5′-CTGGACCGGAGAGGGATG-3′

reverse, 5′-AGCCATAGTTGTTGGCCTGA′

mouse EpCAM forward, 5′-ATCGCTGTCATTGTGGTGGT-3′

reverse, 5′-ACCCATCTCCTTTATCTCAGCC-3′

mouse OCT-4 forward, 5′-CAGTGGGGCGGTTTTGAGT-3′

reverse, 5′-GGCTGAACACCTTTCCAAAGAGA-3′

mouse CD44 forward, 5′-CACCTTGGCCACCACTCCTAAT-3′

reverse, 5′-GATGGTTGTTGTGGGCCGAA-3′

Lentivirus generation and infection

Lentivirus was purchased from Hanheng Biological Technology Co., Ltd (Hanbio, Shanghai, China). The virus titers were all ⩾10^9 TU/ml. We used the 1/2 small volume infection method to explore the most suitable MOI for follow-up experiments. 4T1 cells were cultured in a 12-well plate. When the cell confluence rate reached 40%, 500μl medium was added to each well after washing with PBS, and then 2 μg/ml polybrene has been adjuncted before 10, 15, and 20μl of virus were added respectively. After 72 hours of culture, cellular RNA was extracted for RT-PCR detection to analyze infection efficiency and optimal MOI value. All lentiviruses were designed with three siRNA sequences and synthesized three shRNAs. The most suitable sequences were verified and selected for subsequent experiments. In the subsequent culture and experiments of lentivirus-infected cells, 2μg/ml puromycin was added from time to time to construct a stable lentivirus strain.

Isolation of neutrophils

Mouse neutrophils were collected from female BALB/c mice. Neutrophil isolation kit (2013NM, TBD, Tianjin, China) was used to extract and isolate neutrophils from mouse femur and tibia. Use homogenate to rinse out the lumen. After blowing and beating into a single cell suspension, filter with a 70um filter. Centrifuge at 500g for 10min, resuspend the cells with erythrocyte sedimentation solution, and add it to the separation

solution of the concentration gradient. 800g, centrifuge for 30min. Take the middle cell layer and add 3 times its volume of washing solution to resuspend. Centrifuge at 400g for min. Add erythrocyte lysis solution to remove red blood cells. After centrifugation, add washing solution to wash and centrifuge again to obtain neutrophil pellet. Add appropriate amount of DMEM medium to resuspend, and follow-up experiments can be carried out.

Wound-Healing assay

Tumor cells were culture in 6-well plant, until cell density reached to 90%. 10μl pipette tip was used to scar the cells. Cell wound healing ability was calculated using a Leica microscope at 0h, 12h and 24h. All assays were conducted three times.

Immunohistochemical staining

Briefly, the slide is dewaxed, hydrated, quenched endogenous peroxidase activity, recovered antigen, blocked and combined with anti-HMGCR (1:100, #271595, Santa cruz), anti-CCDC25 (1:100, #515201,Cell Signaling Technology), anti-MPO (1:200, #AF3667, R&D), anti-H3cit (1:300, #4499, Cell Signaling Technology), or anti-Caveolin-1 antibody (1:100, A4480, Cell Signaling Technology) overnight at 4 ℃. Then rinsed the sections, added separated secondary antibodies labeled with horseradish peroxidase, and incubate at 37 ℃ for 1 h. After washing 3 times, use the diaminobenzidine colorimetric reagent solution from Dako (Carpinteria, CA). Subsequently, the slides were counterstained with hematoxylin and dehydrated in graded alcohol and mounted. The expression of CCDC25, Caveolin-1, HMGCR, MPO and H3cit was scored according to the signal intensity and distribution. We used a two-

level scoring method, staining degree 0-3 points (negative, light yellow, light brown, tan), and positive range 1-4 points (0-25%, 26%-50%, 51%-75%, 76%-100 %). The staining degree points were multiplied with positive range points to produce a final score for each case. Tissues with immunohistochemical scoring 2 or less were considered as low, 3 to 12 as high. The immunostaining evaluation was performed independently by two experienced pathologists.

Measurement of serum MPO-DNA level

Anti-MPO monoclonal antibody (5μg/ml, R&D) was coated on a 96-well plate overnight at 4℃. Blocked with 1% BSA for 1h at room temperature then added 100μl serum diluted at 1:5 with normal saline, incubate at room temperature for 2h. After washed with PBST for five times, PicoGreen® dsDNA Quantitation Reagent (YEASEN, Shanghai, China) was used to measure MPO-DNA content based on the manufacturer's protocol. Data were normalized to healthy mouse serum for each test. All assays were conducted at least 3 times.

Supplementary Figures

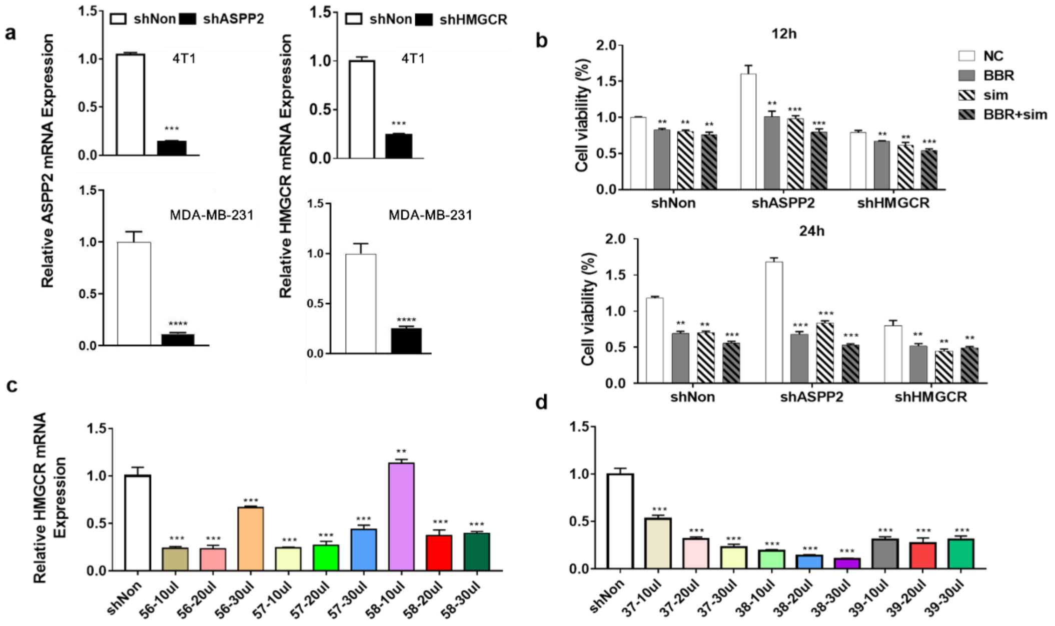

Fig.S1 a 4T1 and MDA-MB-231cells cultured in 6-well plate to verify the efficiency of lentiviral interference with ASPP2 and HMGCR. **b** 4T1 cell treated with 2μM Simvastatin, 10μM Berberine, 2μM Simvastatin and 10μM Berberine or DMSO in 12 and 24 hours for viability assay. **c-d** LV-shHMGCR (c) and LV-shASPP2 (d) lentiviral sequence screening and optimal MOI. Finally, we selected the LV-shHMGCR lentivirus sequence number 56 or LV-shASPP2 lentiviral sequence 38 to infect 4T1 cells. When infecting in a six-well plate, add 20μl lentivirus solution per well. $**P < 0.01$; $***P < 0.001$.

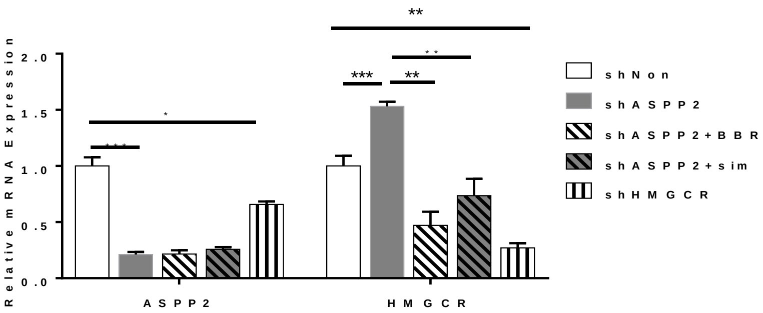

Fig. S2 Analysis of ASPP2 and HMGCR mRNA expression by qRT-PCR in 4T1 cells by different treatment.

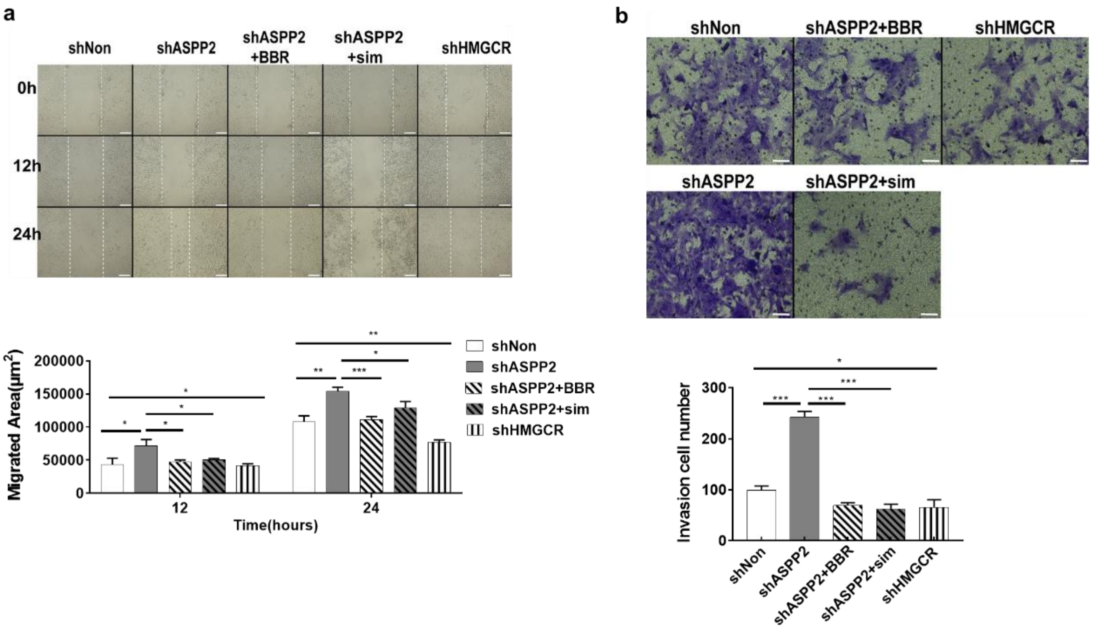

Fig. S3 **a** Wound healing assay (top) and migration areas (down) of treated 4T1 cells. **b** 4T1 cells cultured with Matrigel in the upper chamber of the Transwells. 10μM BBR, 2μM simvastatin or DMSO was added to shASPP2 4T1 cells. 20% serum containing medium were add to the bottom wells. After cultured 22-26h, counted invading cells by a light Microscope. Invasion was calculated by compared with invading cells in shNon group. Scale bar: 200μm.

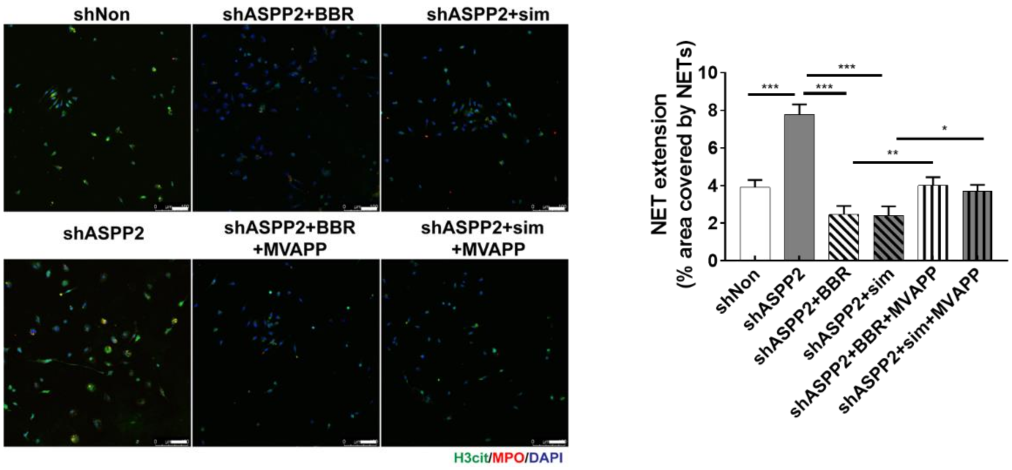

Fig. S4 NETs formation was analyzed after adding MVA pathway additives MVAPP to BBR and simvastatin treated 4T1 cells to recover cholesterol metabolism levels. Scale bar: 50μm. **P* < 0.05; ** *P* < 0.01;*** *P* < 0.001.

Supplementary Tables

Supplementary Table1

The clinicopathologic characteristics of 60 breast cancer patients

**Variables**	**No.of patients(%)**
**Gender**	
Male	0 (0)
Female	60(100)
**Age (year)**	
**<50**	16(27)
**≥50**	44(73)
**AJCC stage**	
Ⅰ-Ⅱ	17(28)
Ⅲ-Ⅳ	27(45)
**Tissue classification**	
Normal or paracancerous tissue	11(18)
Primary breast cancer	27(45)
Metastatic tissue	17(28)
Tumor-free lymph nodes	5 (8)
**Primary tumor with metastasis**	
Yes	24(87)
No	3(13)
**metastatic organ**	
Liver	3(27)
Breast	5(45)
other	3(27)

Supplementary Table2

The HMGCR、CCDC25 and H3cit expression with clinicopathologic characteristics in 27 breast cancer patients

	Whole study(n=27)			Whole study(n=27)			Whole study(n=27)		
	HMGCR expression		*P*	CCDC25 expression		*P*	H3cit expression		*P*
	negative (n=9)	positive (n=18）		negative (n=7)	positive (n=20)		negative (n=6)	positive (n=21)	
Age(years)									
<50	3	6	1.00	4	5	0.175	3	6	0.367
≥50	6	12		3	15		3	15	
Lymph node metastasis number									
<5	6	8	0.42	4	10	1.00	4	10	0.648
≥5	3	10		3	10		2	11	
TNM Stage									
Ⅰ	3	4	0.387	1	6	0.544	3	4	0.395
Ⅱ	3	7		4	6		1	9	
Ⅲ	3	4		1	6		1	6	
Ⅳ	0	3		1	2		1	2	
